# Nitric oxide modulates folate-mediated one-carbon metabolism and mitochondrial energy levels of peaches during cold storage

**DOI:** 10.3389/fnut.2023.1184736

**Published:** 2023-05-05

**Authors:** Zhifeng Yang, Shuhua Zhu, Xiaoyu Wang, Changbao Chen, Dandan Huang, Jianrong Feng

**Affiliations:** ^1^Key Laboratory of Special Fruits and Vegetables Cultivation Physiology and Germplasm Resources Utilization of Xinjiang Production and Construction Crops, Department of Horticulture, Agricultural College, Shihezi University, Shihezi, China; ^2^College of Chemistry and Material Science, Shandong Agricultural University, Taian, China

**Keywords:** nitric oxide, folate, one-carbon metabolism, peach, storage, mitochondria

## Abstract

Folate-mediated one-carbon metabolism (FOCM) is closely associated with postharvest preservation. This study investigated the effects of exogenous nitric oxide (NO) on FOCM, storage quality, energy metabolism, and mitochondrial membrane integrity in cold-storage peach fruit. In this experiment, peaches were soaked with 1.5 mmol L^−1^*S*-nitrosoglutathione (GSNO) as NO donor, and the negative treatment (NT) solution containing 5 μmol L^−1^ carboxy-PTIO (c-PTIO, NO scavenger), 200 μmol L^−1^ N^G^-Nitro-_L_-arginine methyl ester (_L_-NAME, NO synthase-like enzyme inhibitor), and 200 μmol L^−1^ sodium tungstate dihydrate (nitrate reductase inhibitor) and stored at 0°C. The results showed that NO decreased the activity of *S*-adenosylmethionine synthase and *S*-adenosylhomocysteine hydrolase and increased the activity of methionine sulfoxide reductase A, as well as the content of N5-methyl-THF, the ratio of tetrahydrofolate (THF), homocysteine, methionine, *S*-adenosylmethionine (SAM), and SAM to *S*-adenosylhomocysteine compared with the control, indicating that NO effectively increased FOCM flux by affecting the activity of FOCM enzymes. Meanwhile, NO increased the activities of H^+^-ATPase, Ca^2+^-ATPase, cytochrome c oxidase, succinate dehydrogenase, and the contents of adenosine triphosphate and adenosine diphosphate, and maintained high energy charge in peaches during storage. NO retarded the increase in mitochondrial permeability transition, reactive oxygen species content, and the decrease in mitochondrial membrane fluidity, membrane potential, and swelling. NT treatment exhibited the opposite results. In conclusion, these results suggested that NO could induce the accumulation of folate and FOCM flux and maintain mitochondrial energy levels, which might be responsible for maintaining the quality of peaches during cold storage.

## 1. Introduction

Folate, a water-soluble B vitamin, mediates one-carbon metabolism (OCM) and plays a vital role in human health (1). Folate-mediated OCM (FOCM) is a crucial process that provides one-carbon groups for various bioprocesses that are essential for cell survival and proliferation in plants (2). N^5^-methyl-THF (5MTHF), tetrahydrofolate (THF), homocysteine (Hcy), methionine (Met), *S*-adenosylmethionine (SAM), and *S*-adenosylhomocysteine (SAH) are essential substances that are involved in FOCM. Methionine synthase (MetH), *S*-adenosylmethionine synthetase (SAMS), *S*-adenosylhomocysteine hydrolase (SAHH), and methionine sulfoxide reductase (MSR) are the key enzymes involved in this cycle (3). Exogenous THF reduces the rates of weight loss and respiration, inhibits the production of ethylene and reactive oxygen species (ROS), and promotes the activity of antioxidant enzymes and the accumulation of antioxidant substances in broccoli (4). Exogenous Met alleviates cold stress and delays the browning of litchi fruit and broccoli during storage (5, 6). These findings suggest that FOCM plays an essential role in the postharvest preservation of fruits and vegetables.

Low-temperature storage is primarily used to extend the shelf life of fruits (7). In transgenic tobacco, SAMS-derived SAM is preferentially used for polyamine synthesis and homeostasis *in vivo* during cold domestication; this indicates that changes in SAM may be an early plant response to cold stress (8). The THF content in potatoes stored at low temperatures increased gradually with the storage time (9). During the postharvest storage of Honeycrisp fruits, the amino acid content, represented by Met, varied more with temperature than the sugar and organic acid contents (10); this suggests that changes in plant FOCM is a plant response to low temperatures. Nitric oxide (NO) is a reactive oxide of nitrogen that is ubiquitous in living organisms and plays essential roles in various biological processes, including delaying of fruit ripening and improving the quality and shelf life of fruit. NO is a critical gasotransmitter in fruit after harvesting (11). New evidence has recently confirmed the interaction between NO and folate in solution (12). However, information on the regulation of FOCM by NO in postharvest fruit remains scarce.

Peach (*Prunus persica* L. Batsch) is a source of vitamin B and shows spatiotemporal compartmentalization in terms of folate content among varieties, exocarp, mesocarp, and different developmental stages of the fruit (13). In this study, the effects of control (distilled water), NO treatment (exogenous NO donor), and NT treatment (NO inhibitor + NO scavenger) on postharvest storage quality, mitochondrial structure and function, and THF–SAM cycle of peach fruits were pharmacologically investigated to identify the regulation of exogenous NO in FOCM and its role in maintaining the refrigerated quality of peaches after harvesting.

## 2. Materials and methods

### 2.1. Plant materials and treatments

The peaches [*P. persica* (L.) Batsch, cv. Laishanmi] were harvested from a local orchard in Taian, China. *S*-nitrosoglutathione (GSNO), carboxy-PTIO (c-PTIO), N^G^-nitro-_L_-arginine methyl ester (_L_-NAME, NO synthase inhibitor), and sodium tungstate dihydrate (nitrate reductase inhibitor) were bought from Sigma-Aldrich. After precooled overnight, peaches of uniform size and color and without mechanical damage were selected and soaked in distilled water (as control), 1.5 mmol L^−1^ GSNO solution (as NO treatment), and the solution containing 5 μmol L^−1^ c-PTIO, 200 μmol L^−1^_l_-NAME, 200 μmol L^−1^ sodium tungstate dihydrate (as negative treatment, NT), respectively, for 5 min. The concentrations of c-PTIO, _L_-NAME, and sodium tungstate dihydrate were selected based on our previous study (14). Each treatment was repeated three times, with 100 peaches in each replication. After drying with cool air, peaches were stored at 0°C (Haier horizontal refrigerated freezer converter, Model BC/BD-519HEM; relative humidity 70–80%) and sampled once a week.

### 2.2. Measurement of fruit quality

Firmness, the total color difference (ΔE), weight loss rate (WLR), electrolyte leakage (EL), and respiration rate (RR) of peaches were determined according to Wang et al. (15).

### 2.3. Measurement of mitochondrial membrane integrity and energy metabolism

Mitochondria were extracted and purified by sucrose density gradient centrifugation (15). The purified mitochondrial precipitate was resuspended in 100 mmol L^−1^ Tris–HCl (pH 8.5). Protein was quantified with Coomassie brilliant blue (16).

Mitochondrial membrane potential (MMP) was determined using a Cary Eclipse spectrofluorometer (Varian, America) (17). The reaction solution contained 0.4 ml mitochondria, 2 ml 10 mmol L^−1^ Hepes-HCl (pH 7.4, containing 250 mmol L^−1^ sucrose, 2 mmol L^−1^ MgCl_2_, 4 mmol L^−1^ KH_2_PO_4_, 100 μmol L^−1^ K-EGTA). The fluorescence changes at E_x_/E_m_ = 503/527 nm were measured. MMP was expressed as (∆F/Fi) s^−1^ mg^−1^ (in protein).

Mitochondrial membrane fluidity (MMF) was determined using a Cary Eclipse spectrofluorometer (Varian, America) (15). The reaction solution contained 0.1 ml mitochondria, 1.88 ml of 0.3 mol L^−1^ mannitol (The solution pH was adjusted to 7.2 with 0.5 mmol-1 l KOH using a PHS-3C pH meter (Rex Electric Chemical, Shanghai)), and 20 μl 5 mmol L^−1^ 1-anilino-8-naphthalene (ANS). The fluorescence changes at *E*_x_/*E*_m_ = 400/480 nm were measured. MMF was expressed as F mg^−1^ (in protein).

Mitochondrial permeability transition (MPT) was measured using a UV-2450 ultraviolet and visible spectrophotometer (Shimadzu, Japan) (17). The reaction solution contained 0.1 ml mitochondria and 1.9 ml 10.0 mmol L^−1^ Tris–HCl (pH 7.4, containing 125.0 mmol L^−1^ sucrose, 65.0 mmol L^−1^ KCl, 5.0 mmol L^−1^ sodium succinate, 5.0 μmol L^−1^ rotenone,). The change of absorbance at 540 nm was recorded. MPT was expressed as ∆F/F mg^−1^ (in protein).

Mitochondrial swelling (MS) was measured using a UV-2450 ultraviolet and visible spectrophotometer (Shimadzu, Japan) (18). The reaction solution contained 1 ml mitochondria, 0.2 ml of 0.5 mmol L^−1^ FeSO_4_, and 0.2 ml of 0.5 mmol L^−1^ ascorbic acid. The absorbance was immediately detected at 520 nm.

Mitochondrial ROS content (MROS) was determined using a Cary Eclipse spectrofluorometer (Varian, America) (17). Mitochondria (100 μl) was mixed with 900 μl of 10 mmol L^−1^ Tris–HCl (pH 7.2) and 10 μl of 2′,7′-dichlorofluorescein ethylenediolate (DCF-DA), and the changes of fluorescence at E_x_/E_m_ = 485/530 nm were measured. The MROS content was expressed as Arbitrary units mg^−1^ (in protein).

The activities of mitochondrial H^+^-ATPase, Ca^2+^-ATPase, cytochrome c oxidase (CCO), and succinate dehydrogenase (SDH) were measured using a UV-2450 spectrophotometer (Shimadzu, Japan). One unit (U) of the enzymatic activity was defined as the amount of enzyme converting 1 μmol of a substrate within 1 min. The enzymatic activity was expressed as U mg^−1^ (in protein).

Mitochondrial H^+^-ATPase and Ca^2+^-ATPase activities were determined according to Ren, Zhu (19). Mitochondrial resuspension (0.15 ml) was added to 1 ml of 30 mmol L^−1^ Tris–HCl buffer (pH 7.5, containing 50 mmol L^−1^ KCl and 3 mmol L^−1^ MgSO_4_ or 10 mmol L^−1^ CaCl_2_), 0.1 ml of 3 mmol L^−1^ adenosine triphosphate (ATP) was added to initiate the reaction, and the reaction was stopped at 37°C for 30 min, followed by 0.2 ml of 30% trichloroacetic acid (TCA; w/v). The absorbance at 660 nm was measured.

The activity of CCO was determined by the method of Kan et al. (20). Mitochondria (0.15 ml) was mixed with 0.2 ml of 0.45 mmol L^−1^ cytochrome c solution (cytochrome c was first configured as 0.45 mmol L^−1^ aqueous solution by adding 200 mg/ml of _L_-ascorbic acid to reduce cytochrome c to A550 (reduced state)/A565 (oxidized state) >12) and 2 ml of buffer (pH 7.4, containing 200 mmol L^−1^ K_3_PO_4_ and 2% TritonX-100 (w/v)). The absorbance was measured at 550 nm.

Mitochondrial SDH enzyme activity was determined as described by Ackrell et al. (21). The mitochondria (0.15 ml) were incubated at 30°C for 5 min in a 4.1 ml reaction solution containing 3 ml of 0.2 mol L^−1^ phosphate buffer (pH 7.4), 1 ml of 0.2 mol L^−1^ sodium succinate (pH 7.4) and 0.1 ml of 1 mmol L^−1^ sodium 2, 6-dichlorophenol indigo (DCPIP). The reaction was started by adding 0.33% methylthiophenazine (w/v; 0.1 ml). The rate of reduction of DCPIP at 600 nm was measured.

High performance liquid chromatography (HPLC; LC-20A, Shimadzu, Japan) equipped with a Kromasil C18 column (250 × 4.6 mm, 5 μm) was used to determine ATP, adenosine diphosphate (ADP), and adenosine monophosphate (AMP) content (22). The sample (1 g) was mixed with 3 ml of 0.6 mol L^−1^ perchloric acid and then centrifuged (16,000 × *g*, 4°C) for 30 min. Quickly neutralized 1.5 ml of supernatant with 1 mol L^−1^ KOH to pH 6.5–6.8 and then passed through the 0.45 μm membrane filter. Mobile phase A was a solution of 60 mmol L^−1^ K_2_HPO_4_ and 40 mmol L^−1^ KH_2_PO_4_ (pH 7.0), and mobile phase B was 100% methanol (v/v). The flow rate was 1 ml min^−1^, with a gradient program as follows: 0–7 min, 0–20% B (v/v); 7–9 min, 20–25% B (v/v); 9–10 min, 25–0% B (v/v); 10–12 min, 0% B (v/v). The injection volume was 20 μl, and the wavelength at 254 nm. The adenosine energy charge (EC) was calculated as: [(ATP) + 0.5 × (ADP)]/[(ATP) + (ADP) + (AMP)].

### 2.4. Measurement of parameters in folate-mediated one-carbon metabolism

The measurement of MetH activity was based on the method described by Grabowski et al. (23). The sample (1 g) was homogenized on ice with 2 ml of 20 mmol L^−1^ 4-(2-Hydroxyethyl)-1-piperazine ethanesulfonic acid (Hepes; containing 14 mmol L^−1^ NaCl, 3 mmol L^−1^ MgCl_2_, 5% glycerol (w/v), 0.5% Igepal CA-630 (w/v), 1 mmol L^−1^ dithiothreitol (DTT), 1 mmol L^−1^ phenylmethylsulfonyl fluoride), and then centrifuged (12,000 × *g*, 4°C) for 20 min. The enzyme extract (0.2 ml) was added to 0.8 ml of 1 mol L^−1^ phosphate buffer (containing 0.02 ml of 1 mol L^−1^ DTT, 0.048 ml of 4.2 mmol L^−1^ THF, and 0.02 ml of 0.76 mmol L^−1^ SAM). The resulting test mixture was added to 0.08 ml of 0.5 mmol L^−1^ hydroxocobalamin, and the mixture was immediately pre-incubated for 5 min at 37°C. After initiation with 0.004 ml of 100 mmol L^−1^_l_-homocysteine, the reaction was incubated for 10 min at 37°C and then terminated with 0.2 ml of 5 mmol L^−1^ HCl/60% formic acid (w/v) and incubated for 10 min at 80°C. The change in absorbance at 350 nm was recorded.

*S*-adenosylmethionine synthetase activity was measured as described by Wang et al. (24). The sample (1 g) was homogenized on ice with 2 ml of enzyme extraction buffer containing 50 mmol L^−1^ Tris–HCl (pH 7.6), 5 mmol L^−1^ 2-mercaptoethanol, 10 mmol L^−1^ MgCl_2_, 0.1 mmol L^−1^ ethylene diamine tetraacetic acid (EDTA) and 2% polyvinylpyrrolidone (w/v), and then centrifuged (10,000 × *g*, 4°C) for 30 min. The enzyme extract (0.4 ml) was mixed with 0.6 ml of 100 mmol L^−1^ Tris–HCl (pH 8.0, containing 20 mmol L^−1^ MgCl_2_, 150 mmol L^−1^ KCl, 2 mmol L^−1^ ATP, 5 mmol L^−1^ dithiothreitol and 1 mmol L^−1^ methionine). The absorbance at 340 nm was measured.

SAHH activity was measured based on Yang et al. (25). The sample (1 g) was homogenized on ice with 2 ml of precooled enzyme extraction buffer (50 mmol L^−1^ Hepes, pH 7.8, 5 mmol L^−1^ DTT, 1 mmol L^−1^ Na_2_EDTA, 5 mmol L^−1^ ascorbic acid, 10 mmol L^−1^ boric acids, 20 mmol L^−1^ sodium metabisulphate and 4% polyvinylpyrrolidone (w/v)), and then centrifuged (12,000 × *g*, 4°C) for 20 min. The supernatant (0.2 ml) was mixed with 0.8 ml 50 mmol L^−1^ HEPES-KOH (pH 7.8, containing 0.1 mmol L^−1^ 5,5′-Dithiobis-(2-nitrobenzoic acid; DTNB), 0.1 mmol L^−1^ SAH, and 1 mmol L^−1^ EDTA), and incubated for 3 min at 25°C. The change in absorbance at 412 nm was recorded.

Methionine sulfoxide reductase A (MsrA) activity was determined as described by Wu et al. (26). The sample (1 g) was homogenized on ice with 2 ml of 50 mmol L^−1^ potassium phosphate buffer (pH 7.5) containing 0.1 mmol L^−1^ EDTA, 0.3% TritonX-100 (w/v), 4% polyvinylpyrrolidone (PVP; w/v), and then centrifuged (12,000 × *g*, 4°C) for 20 min. The supernatant was collected and used for the enzyme assay. The 0.1 ml supernatant was mixed with 0.4 ml reaction buffer (10 mmol L^−1^ MgCl_2_, 30 mmol L^−1^ KCl, 25 mmol L^−1^ Tris–HCl, 0.5 mmol L^−1^ dimethyl sulfoxide, 0.1 mmol L^−1^ dithiothreitol, pH 8.0) for 30 min at 37°C protected from light. After that, an equal volume of 4 mmol L^−1^ DTNB was added and further incubated at 37°C for 10 min. The change of absorbance at 412 nm was monitored.

The contents of 5MTHF, THF, Hcy, Met, SAM, and SAH were determined using HPLC (Shimadzu LC-20A, Japan) equipped with a Kromasil C-18 column (250 × 4.6 mm, 5 μm) and expressed as mol kg^−1^ (in fresh weight). The measurements of 5MTHF and THF contents were based on Delchier et al. (27). The sample (5 g) was added to 20 ml of extraction buffer containing 50 mmol L^−1^ K_2_HPO_4_, 1 mmol L^−1^ CaCl_2_, 2% ascorbic acid (w/v), 0.1% β-mercaptoethanol (w/v), and reacted at 100°C for 15 min, then centrifuged at 12000 *g* for 20 min. The supernatant was passed through the 0.45 μm membrane filter for HPLC analysis. The mobile phase was 50 mmol L^−1^ KH_2_PO_4_-acetonitrile (92.5:7.5, v/v) with the flow rate at 1 ml min^−1^. The column temperature was 35°C, and the injection volume was 20 μl.

Hcy and Met were separated on a reversed-phase C-18 column using *o*-phthalaldehyde for pre-column derivatization, followed by fluorescence detection (28, 29). The sample (3 g) was added to 22.5 ml of 0.1% HCl (w/v), water bath (100°C) for 12 h, sonication at 200 W for 12 h, centrifugation at 12,000 × *g* for 15 min, and the supernatant was passed through the 0.45 μm membrane filter for HPLC analysis. Mobile phase A was a solution of 20 mmol L^−1^ phosphate buffer (pH 6.5), and mobile phase B was acetonitrile: methanol: double distilled water = 45:40:15 (v/v/v). The excitation and the emission wavelength were 350 nm and 450 nm, respectively. The flow rate was 0.8 ml min^−1^, and the injection volume was 10 μl, with a gradient program as follows: 0–11% B (v/v); 2–4 min, 11–17% B (v/v); 4–5.5 min, 17–31% B (v/v); 5.5–10 min, 31–32.5% B (v/v); 10–12 min, 32.5–46.5% B (v/v); 12–15.5 min, 46.5–55% B (v/v). 15.5–16 min, 55–100% B (v/v); 16–20 min, 100–11% B (v/v); 20–25 min, 11–8% B (v/v); 25–30 min, 8–5% B (v/v); 30–40 min, 5–0% B (v/v).

The SAM and SAH contents were determined as described by She et al. (30). The sample (1.5 g) was mixed with 0.4 mol L^−1^ HClO_4_ for 15 min. The crude extract was centrifuged at 16,400 × *g* for 30 min at 4°C. Then the supernatant was passed through the 0.45 μm membrane filter for HPLC analysis. The mobile phase comprised 40 mmol L^−1^ NH_4_H_2_PO_4_, 8 mmol L^−1^ 1-heptanesulfonic acid sodium salt, and 18% (v/v) methanol (pH 3.0). The column temperature was 35°C, the flow rate was 0.8 ml min^−1^, the injection volume was 50 μl, and the wavelength at 254 nm. The SAM/SAH ratio was calculated as the methylation index (MI), indicating the methylation status.

*S*-nitrosoglutathione reductase (GSNOR) activity was determined by monitoring the oxidation of NADH at 340 nm as described by Sakamoto et al. (31) and expressed as U kg^−1^ (in protein). The sample (1 g) was homogenized on ice with 3 ml of extraction buffer (containing 100 mmol L^−1^ Tris–HCl (pH 8.0), 1 mmol L^−1^ EDTA, 10% glycerol (v/v), 0.1% TritonX-100 (v/v)), and then centrifuged (16,000 × *g*, 4°C) for 15 min. The reaction solution contained 0.4 ml of supernatant and 3.6 ml of the reaction mixture (containing 20 mmol L^−1^ Tris–HCl (pH 8.0), 0.2 mmol L^−1^ NADPH and 0.5 mmol L^−1^ EDTA). The mixture was incubated for 75 s, and the reaction was started by adding 10 μl of 100 mmol L^−1^ GSNO to a final concentration of 400 μmol L^−1^.

### 2.5. Statistical analysis

Each experiment was carried out with three biological replicates. Data were expressed as means ± standard deviations and analyzed using one-way analysis of variance (ANOVA) and Tukey’s test.

## 3. Results

### 3.1. Changes in the quality of peaches

**Figure 1:**
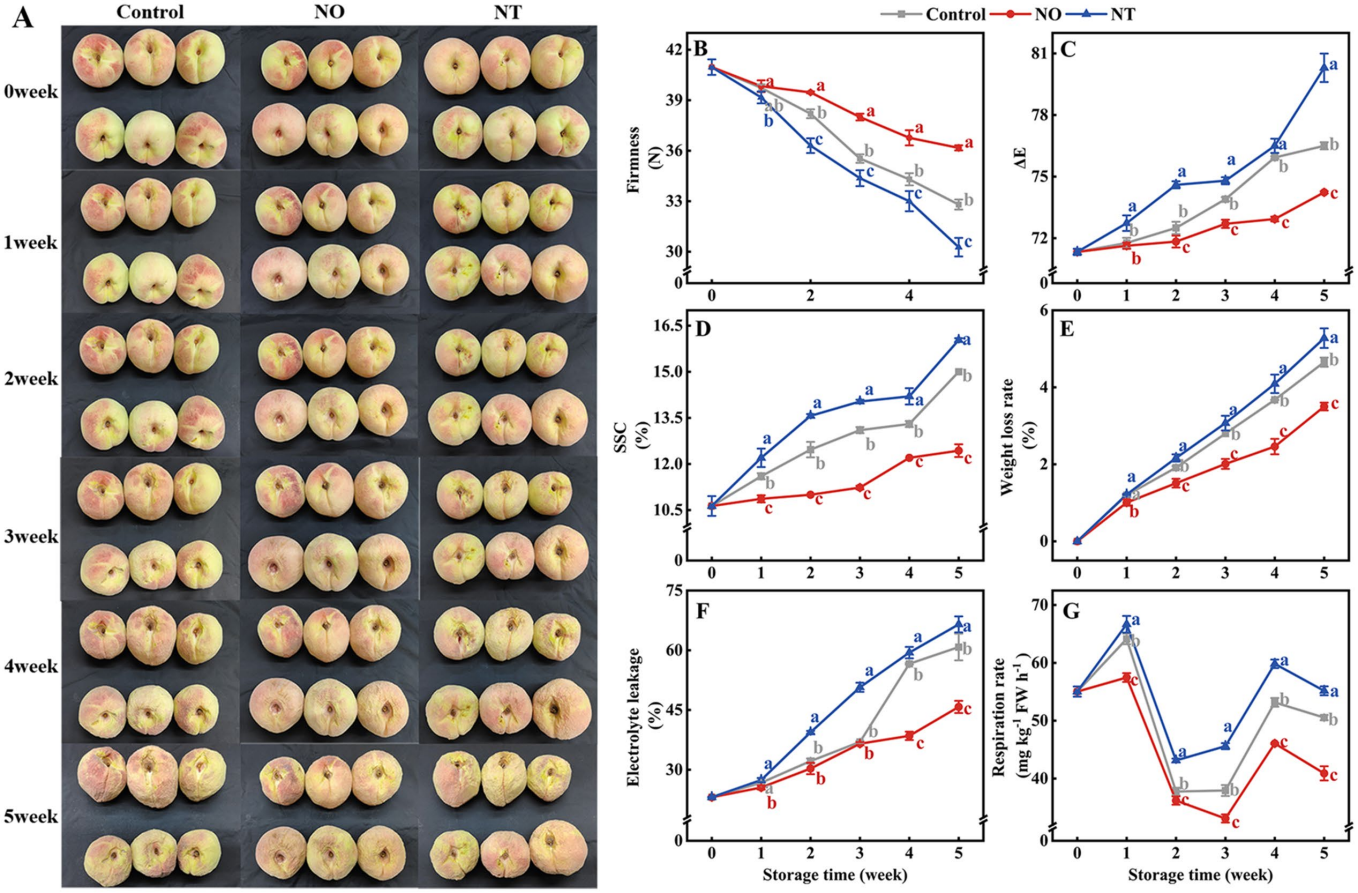
Changes in the appearance **(A)** and quality **(B–G)** of peaches after different treatments and storage at low temperatures (0°C) for 5  weeks. The firmness **(B)**, ΔE **(C)**, soluble solids content (SSC) **(D)**, weight loss rate (WLR) **(E)**, electrolyte leakage (EL) **(F)**, and respiration rate (RR) **(G)** of peaches during storage at 0°C. NO treatment, 1.5  mmol L^−1^ GSNO solution; NT treatment, the solution containing 5  μmol L^−1^ c-PTIO, 200  μmol L^−1^_l_-NAME, 200  μmol L^−1^ sodium tungstate dihydrate. Values represent the means ± SD (*n* = 3). Values with different letters within the same sampling time are significantly different (*p* < 0.05).

With the prolonged low-temperature storage, the peach fruit gradually lost water and wrinkled (Figure 1A). In the third week, the surface of the peach fruit began to show a more obvious water loss and shrinkage. In the fifth week, NO treatment alleviated peach fruit shrinkage compared to the control, while NT treatment exacerbated peach fruit shrinkage.

The ΔE, SSC, WLR, and EL increased over time during cold storage, while the firmness gradually decreased (Figures 1B–F). The two respiration peaks of peaches occurred on the 1st week and the 4th week of storage, respectively (Figure 1G). Compared with the control, NO treatment suppressed the increase in ΔE, SSC, WLR, EL, and RR and alleviated the decrease in firmness, and NT treatment had the opposite phenomenon. In particular, compared with the control, ΔE, SSC, WLR, EL, and RR of peaches treated with NO were significantly (*p* < 0.05) reduced by 2.96, 17.11, 24.79, 24.73, and 19.02% in the 5th week, respectively. The firmness increased significantly (*p* < 0.05) by 10.26% for the NO treatment compared to the control. In contrast, ΔE, SSC, WLR, EL, and RR were significantly (*p* < 0.05) increased by 4.97, 6.89, 13.40, 9.37, and 9.16%, respectively, and firmness was significantly (*p* < 0.05) decreased by 7.72% in NT treatment.

### 3.2. Changes in mitochondrial membrane integrity and energy metabolism

**Figure 2:**
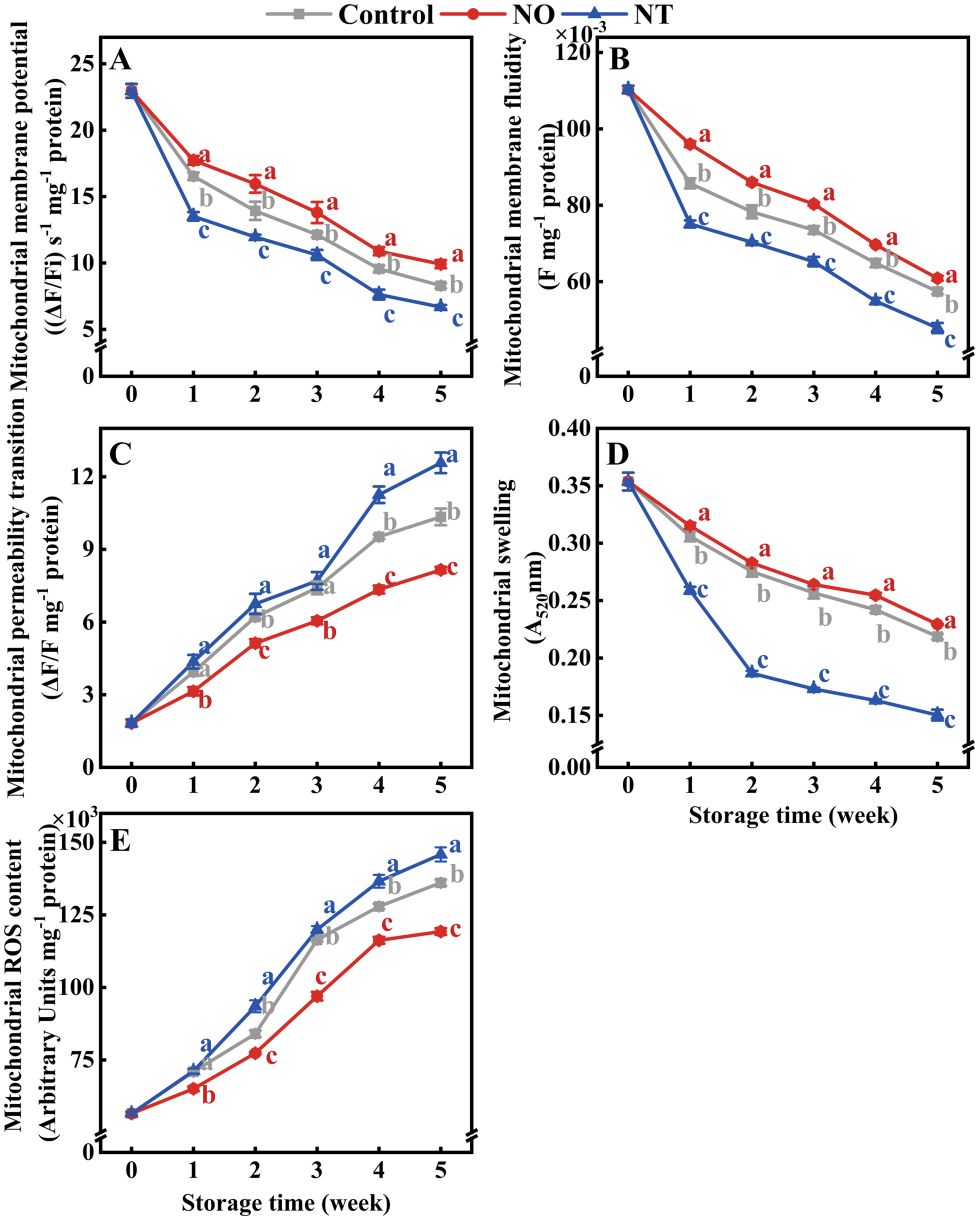
The mitochondrial membrane potential (MMP) **(A)**, mitochondrial membrane fluidity (MMF) **(B)**, mitochondrial permeability transition (MPT) **(C)**, mitochondrial swelling (MS) **(D)**, and mitochondrial ROS content (MROS) **(E)** of peaches during storage at 0°C. Values represent the means ± SD (*n* = 3). NO treatment, 1.5 mmol L^−1^ GSNO solution; NT treatment, the solution containing 5  μmol L^−1^ c-PTIO, 200  μmol L^−1^_l_-NAME, 200  μmol L^−1^ sodium tungstate dihydrate. Values with different letters within the same sampling time are significantly different (*p* < 0.05).

The MMP, MMF, and MS of peaches gradually decreased during cold storage (Figures 2A,B,D), while MPT and MROS gradually increased (Figures 2C,E). Compared with the NO, NT treatment had the opposite effect. In comparison with the control, NO inhibited the increase of MPT and MROS and alleviated the decrease of MMP, MMF, and MS (Figure 2). At week 5, compared to the control, the NO treatment reduced MPT and MROS by 21.15 and 12.35%, and increased MMP, MMF, and MS by 19.63, 6.03, and 4.88%, respectively. In contrast, the NT treatment increased MPT and MROS by 21.59 and 7.18% and decreased MMP, MMF, and MS by 19.14, 16.68, and 31.25%, respectively.

**Figure 3:**
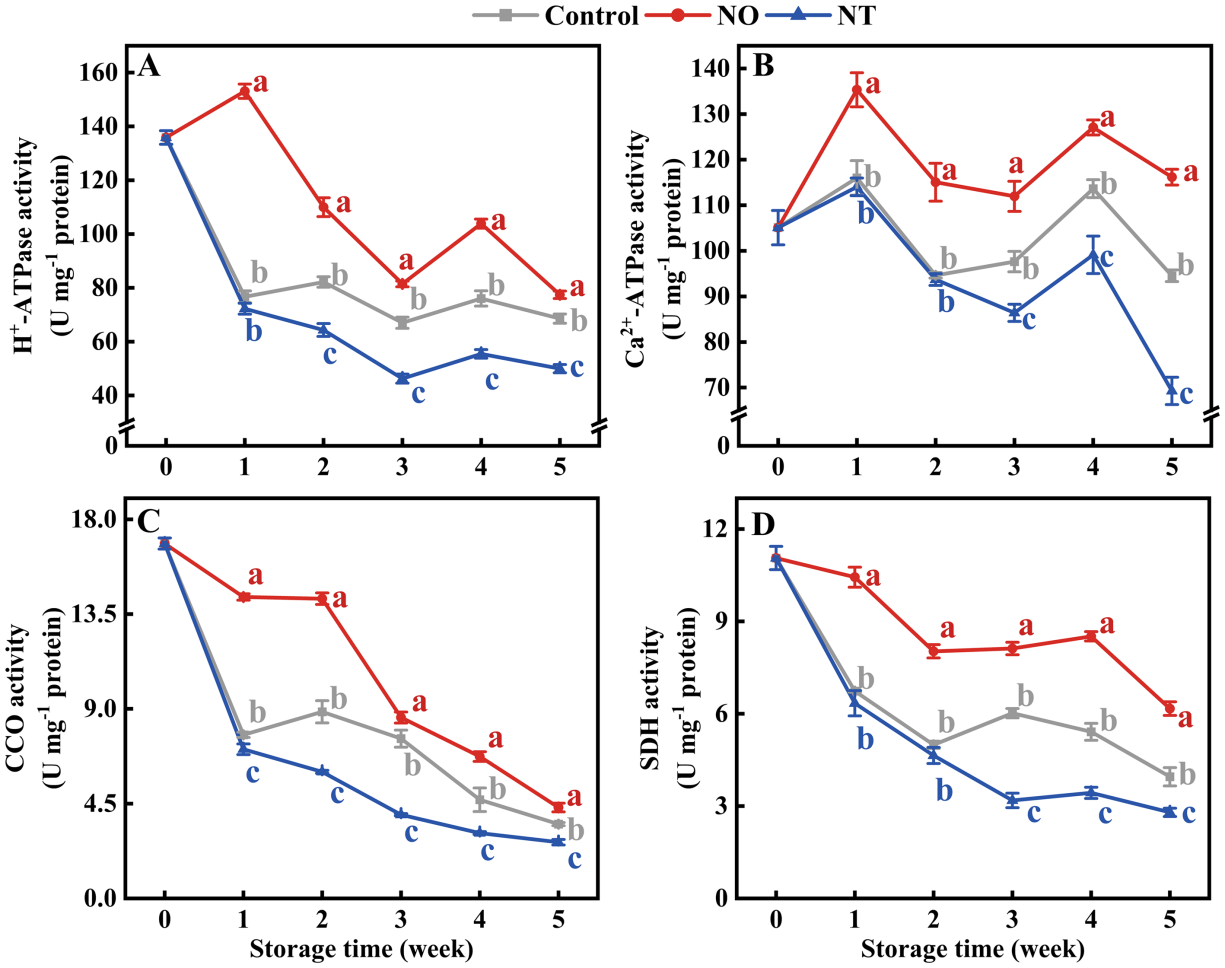
H^+^-ATPase **(A)**, Ca^2+^-ATPase **(B)**, cytochrome c oxidase (CCO) **(C)**, succinate dehydrogenase (SDH) **(D)** of peaches during storage at 0°C. Values represent the means ± SD (*n* = 3). Values with different letters within the same sampling time are significantly different (*p* < 0.05).

The mitochondrial H^+^-ATPase, Ca^2+^-ATPase, CCO, and SDH enzymes activities of peaches were gradually reduced during storage (Figures 3A–D). Compared with the control, NO treatment inhibited the decline of H^+^-ATPase, Ca^2+^-ATPase, CCO, and SDH enzyme activities, while the NT had the opposite effect. The H ^+^-ATPase and Ca^2+^-ATPase enzyme activities of peaches peaked at week 4 (Figures 3A,B). In particular, the H^+^-ATPase and Ca^2+^-ATPase activities of NO-treated peaches peaked at week 1. Additionally, compared to the control, NO treatment significantly (*p* < 0.05) delayed the descent of H^+^-ATPase, Ca^2+^-ATPase, CCO, and SDH activities by 99.57, 16.63, 84.32, and 55.00%, NO treatment significantly (*p* < 0.05) improved H^+^-ATPase, Ca^2+^-ATPase, CCO, and SDH activities by 27.08, 26.72, 24.32 and 29.28% at week 1, respectively.

**Figure 4:**
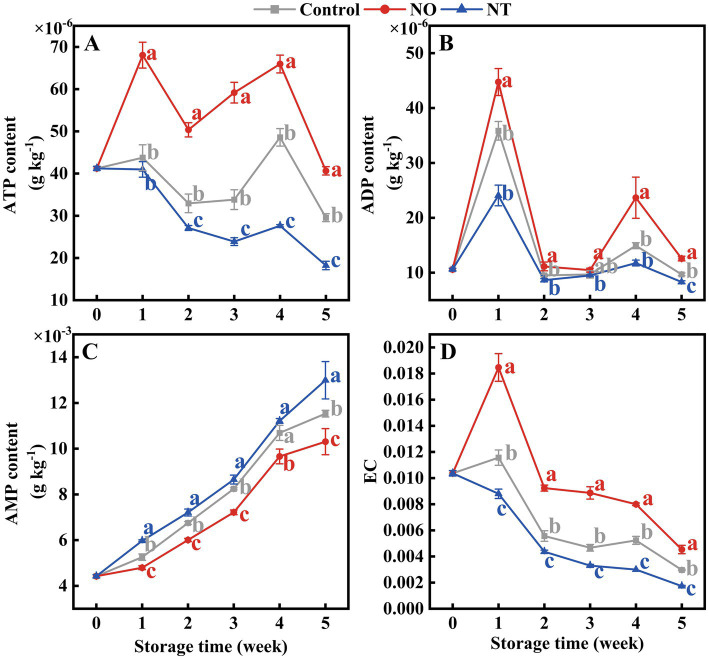
Adenosine triphosphate (ATP) **(A)**, adenosine diphosphate (ADP) **(B)**, adenosine monophosphate (AMP) **(C)**, and energy charge (EC) **(D)** of peaches during storage at 0°C. Values represent the means ± SD (*n* = 3). Values with different letters within the same sampling time are significantly different (*p* < 0.05).

ATP and ADP contents showed peaks at weeks 1 and 4, respectively (Figures 4A,B). In contrast, AMP gradually accumulated during storage (Figure 4C). At week 5, compared to the control, the ATP, ADP, and EC content of peaches (Figures 4A,B,D) treated with NO were significantly (*p* < 0.05) increased by 37.52, 29.88, and 52.81%, and that of NT treatment significantly (*p* < 0.05) decreased by 38.33, 14.35, and 41.57%, respectively.

### 3.3. Changes in folate-mediated one-carbon metabolism and GSNOR activity

**Figure 5:**
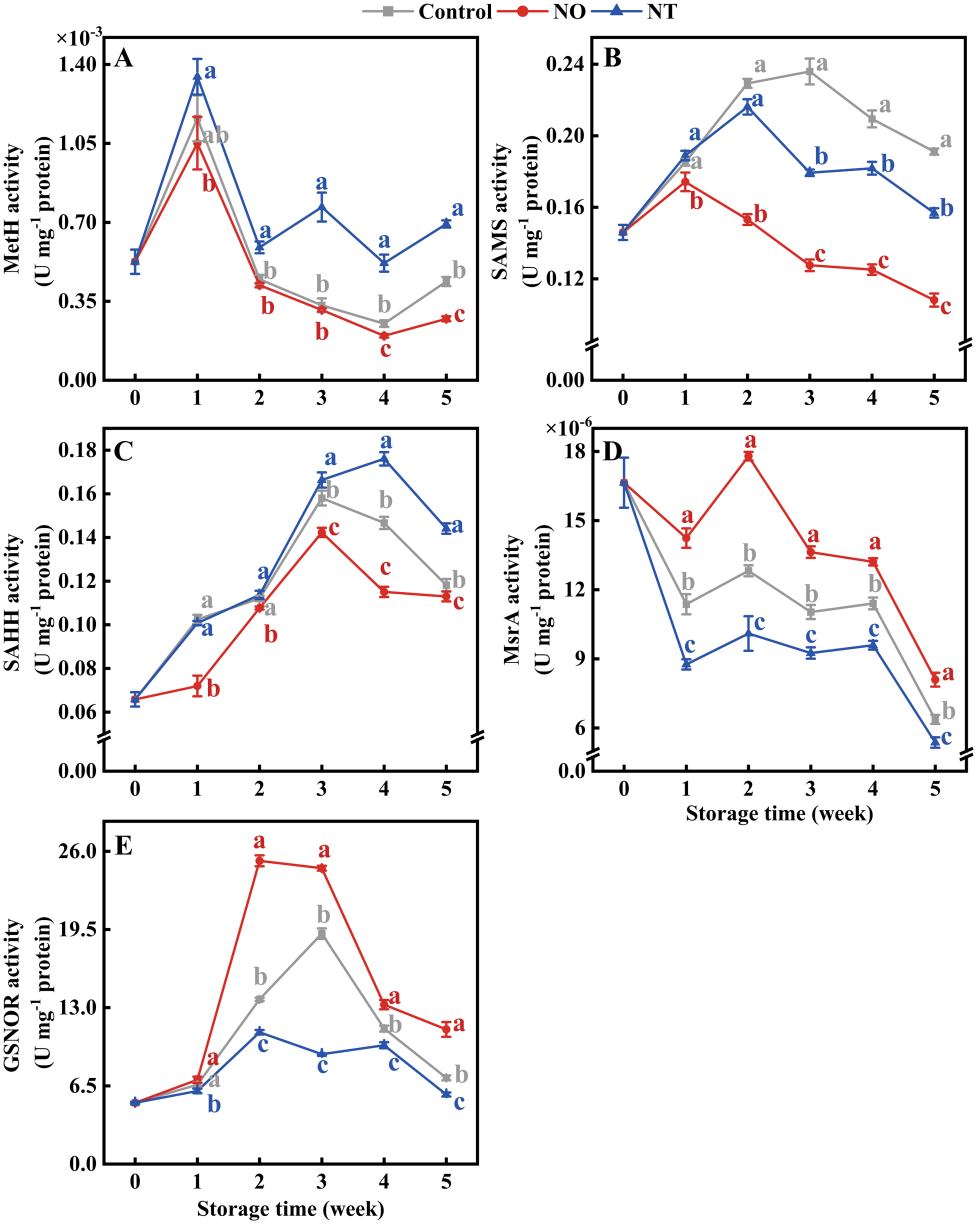
Methionine synthase (MetH) **(A)**, *S*-adenosylmethionine synthetases (SAMS) **(B)**, *S*-adenosylhomocysteine hydrolase (SAHH) **(C)**, methionine sulfoxide reductases A (MsrA) **(D)**, and *S*-nitrosoglutathione reductase (GSNOR) **(E)** of peaches during storage at 0°C. Values represent the means ± SD (*n* = 3). Values with different letters within the same sampling time are significantly different (*p* < 0.05).

Over time, MetH, SAMS, and SAHH showed a trend of increasing and then decreasing (Figures 5A–C). MetH in NT treatment was higher (*p* < 0.05) than the control after week 2 (Figure 5A). SAMS and SAHH were effectively reduced by NO treatment (Figures 5B,C). In contrast, MsrA was significantly (*p* < 0.05) elevated by NO treatment (Figure 5D). At week 5, NO-treated MetH, SAMS, SAHH, and MsrA were 0.62, 0.57, 0.96, and 1.27 times higher than the control, respectively. NT-treated MetH, SAMS, SAHH, and MsrA were 1.58, 0.82, 1.22, and 0.84 times higher than the control, respectively (Figures 5A–D).

As shown in Figure 5E, the GSNOR activity of peaches increased and then decreased during storage. At week 2, the GSNOR activity of peaches treated with NO was significantly (*p* < 0.05) increased by 84.08% compared with the control. The peak of NO treatment appeared at week 2 and earlier than the control. NT treatment consistently inhibited GSNOR activity (*p* < 0.05).

**Figure 6:**
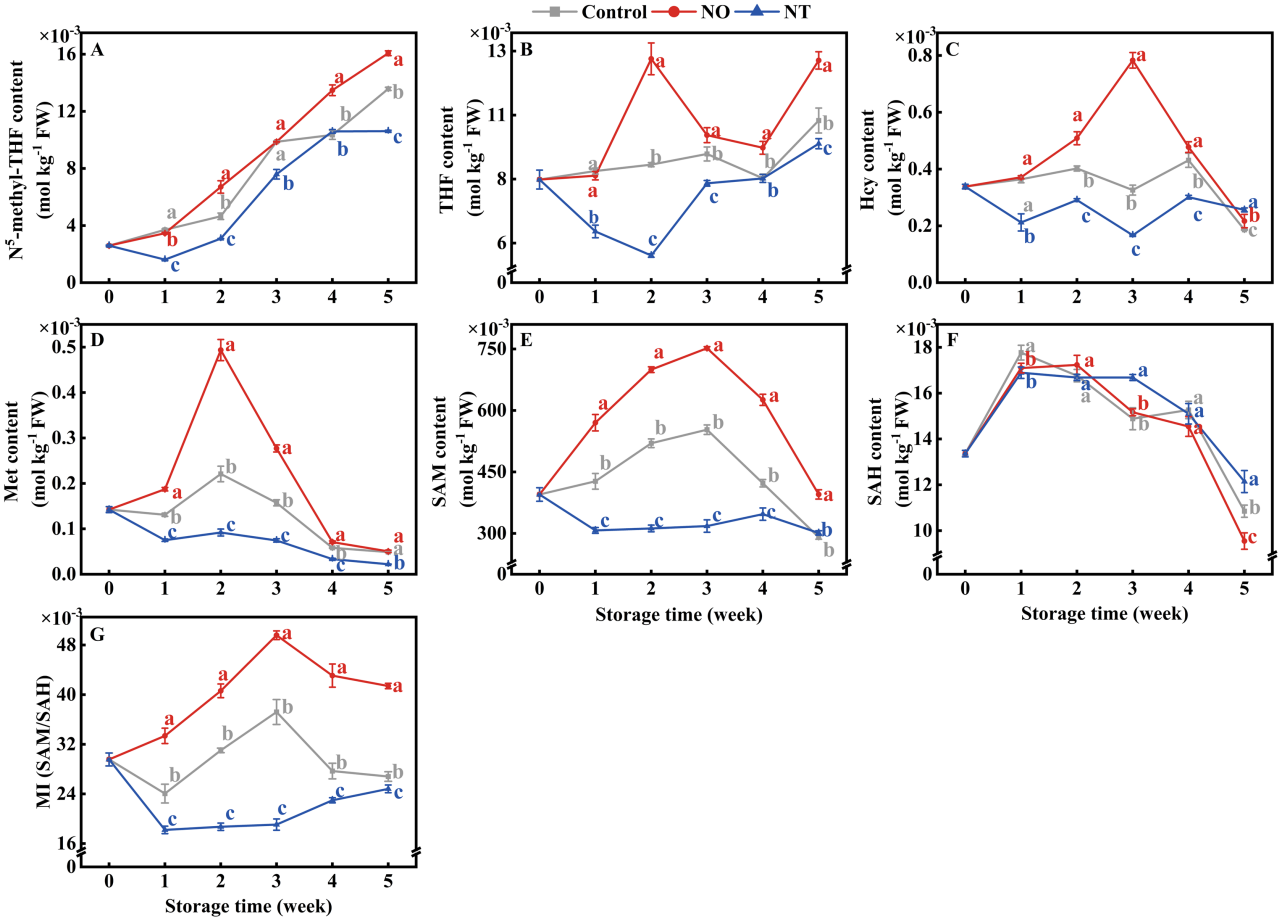
N^5^-methyl-THF (5MTHF) **(A)**, tetrahydrofolate (THF) **(B)**, homocysteine (Hcy) **(C)**, methionine (Met) **(D)**, *S*-adenosylmethionine (SAM) **(E)**, *S*-adenosylhomocysteine (SAH) **(F)** and methylation index (MI) **(G)** of peaches during storage at 0°C. Values represent the means ± SD (*n* = 3). Values with different letters within the same sampling time are significantly different (*p* < 0.05).

The contents of 5MTHF and THF accumulated gradually during storage (Figures 6A,B). The Hcy, Met, SAM, SAH, and MI contents of peaches first increased and then decreased during storage. NO treatment alleviated the decrease of Hcy, Met, SAM, and MI compared with the control, while the NT had the opposite effect (Figures 6C–G). In particular, Hcy and Met of peaches peaked at week 2, while SAM and MI peaked at week 3. In the 2nd week, NO-treated peaches of Hcy and Met contents were 1.26 and 2.23 times higher than the control, while NT treatment was 0.72 and 0.42 times that of the control. On week 3, SAM and MI of NO-treated peaches were 1.36 and 1.33 times higher than the control, while NT treatment was 0.57 and 0.51 times that of the control, respectively.

### 3.4. Correlation analysis of measurement indicators

**Figure 7:**
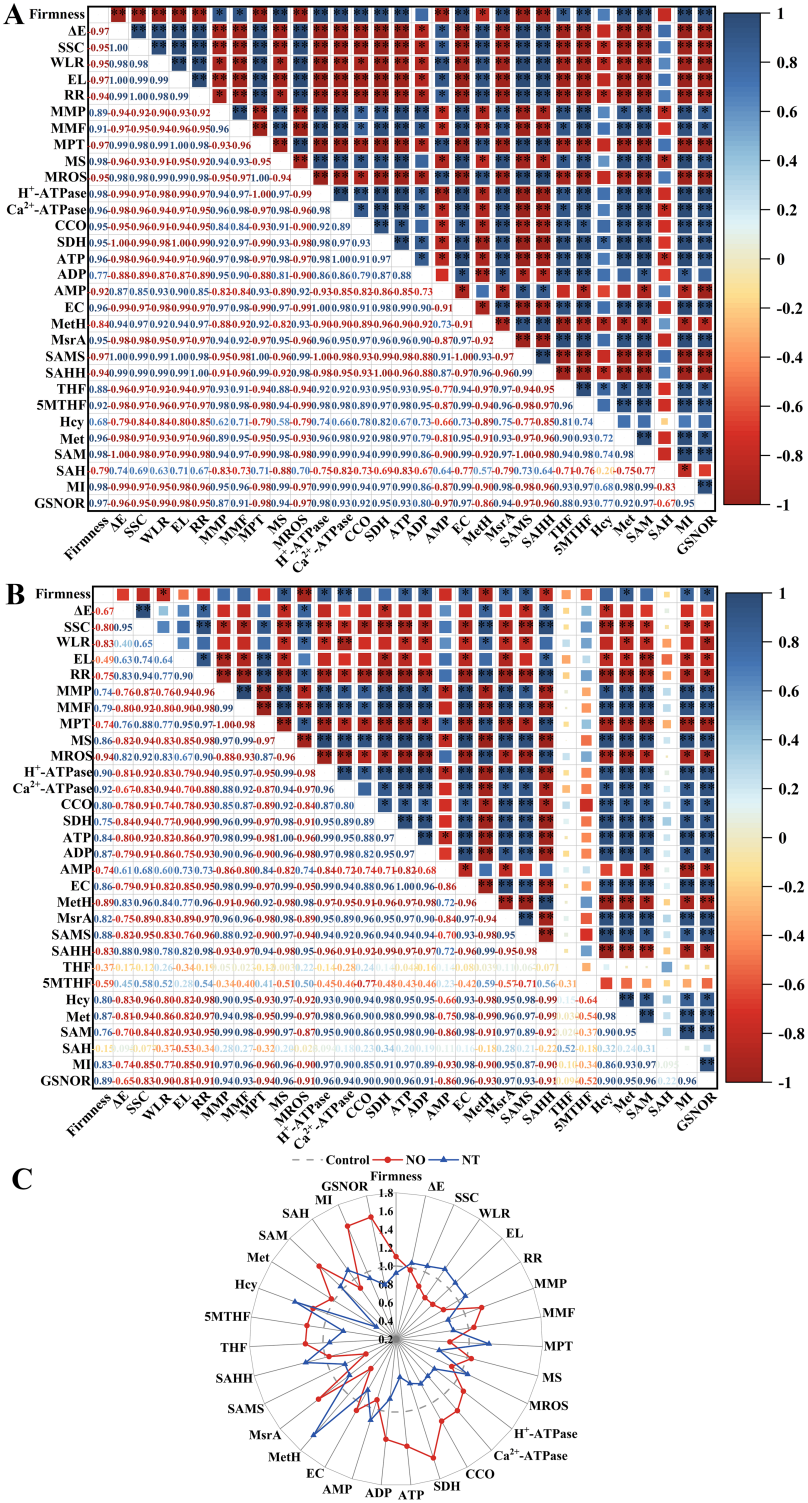
The Pearson correlation coefficient matrix of NO treatment **(A)** and NT treatment **(B)** on postharvest storage quality, mitochondrial structural integrity, energy metabolism-related enzyme activity and substance content, and FOCM-related indexes of peach fruit after 5 weeks of cold storage (0°C). Changes in the above indicators **(C)**. ΔE, total color difference; SSC, soluble solids content; WLR, weight loss; EL, electrolyte leakage; RR, respiration(Continued)FIGURE 7 (Continued) rate; MMP, mitochondrial membrane potential; MMF, mitochondrial membrane fluidity; MPT, mitochondrial permeability transition; MS, mitochondrial swelling; MROS, mitochondrial ROS; CCO, cytochrome c oxidase; SDH, succinate dehydrogenase; ATP, adenosine triphosphate; ADP, adenosine diphosphate; AMP, adenosine monophosphate; EC, energy charge; MetH, methionine synthase; SAMS, *S*-adenosylmethionine synthetases; SAHH, *S*-adenosylhomocysteine hydrolase; MsrA, methionine sulfoxide reductases A; 5MTHF, N^5^-methyl-THF; THF, tetrahydrofolate; Hcy, homocysteine; Met, methionine; SAM, *S*-adenosylmethionine; SAH, *S*-adenosylhomocysteine; MI, methylation index; GSNOR, *S*-nitrosoglutathione reductase. *Indicates a significance level of *p* < 0.05. **Indicates a significance level of *p* < 0.01.

There was a close relationship between quality, mitochondrial membrane structural integrity, energy metabolism, indicators related to the synthesis of methylated methyl donors, and GSNOR on week 5 (Figure 7). For NO treatment, the storage quality (ΔE, SSC, WLR, EL, and RR) was significantly positively (*p* < 0.05) correlated with mitochondrial membrane integrity indicators (MMP, MMF, and MS), energy metabolism-related indicators (H^+^-ATPase, Ca^2+^-ATPase, CCO, SDH, ATP, ADP, and EC), and FOCM related parameters (MsrA, THF, 5MTHF, MI) and GSNOR. MI significantly (*p* < 0.01) negatively correlated with firmness, mitochondrial membrane integrity indicators (MMP, MMF, and MS), energy metabolism-related indicators (H^+^-ATPase, Ca^2+^-ATPase, CCO, SDH, ATP, ADP, and EC), FOCM related parameters (MsrA, THF, 5MTHF, Met, and SAM) and GSNOR (Figure 7A). The correlation of MI values with FOCM-related indicators (SAMS, THF, 5MTHF, SAH) showed an opposite trend in the NT treatment compared to the NO treatment (Figures 7A,B).

Compared to the control, NO treatment had more obvious increases in firmness, MMP, MS, H^+^-ATPase, Ca^2+^-ATPase, CCO, SDH, ATP, ADP, EC, MSR, THF, 5MTHF, Hcy, Met, SAM, MI, GSNOR, and more apparent decreases in ΔE, SSC, WLR, EL, RR, MPTP, MROS, AMP, MetH, SAMS, SAHH, SAH. The trend of NT treatment was opposite to that of NO treatment (except SAMS, Hcy, and SAM; Figure 7C).

## 4. Discussion

Exogenous NO significantly maintained fruit quality during cold storage, whereas c-PTIO and sodium tungstate decreased the quality (32). Firmness and ΔE influence the visual judgment of fruit quality. Firmness reflects the fruit’s storage resistance and is related to the change in WLR; ΔE reflects the degree of fruit browning. In this study, the increase of ΔE, SSC, WLR, and EL and the decrease of firmness during storage (Figures 1B–F) indicated that the degree of fruit suffering cold stress depended on the extension of low temperature (0°C) time, which was manifested by the gradual softening of fruits, the onset of browning, the increase of ripeness, the gradual loss of water and the damage of cell membranes. Compared with the control, NO treatment effectively suppressed the increase in ΔE, SSC, WLR, EL, and RR and alleviated the decrease in firmness (Figures 1B–G), indicating that NO could alleviate the cold stress induced decline in quality. In NO-treated peach fruit, FOCM fluxes (THF, 5MTHF, Met, SAM) were all highly significantly and positively correlated with storage quality (ΔE, SSC, WLR, EL, and RR) at week 5. GSNOR was highly significantly or significantly negatively correlated with storage quality (ΔE, SSC, WLR, EL, and RR; Figure 7A). This result suggests that freshness retention *via* postharvest NO treatment is closely related to FOCM.

**Figure 8:**
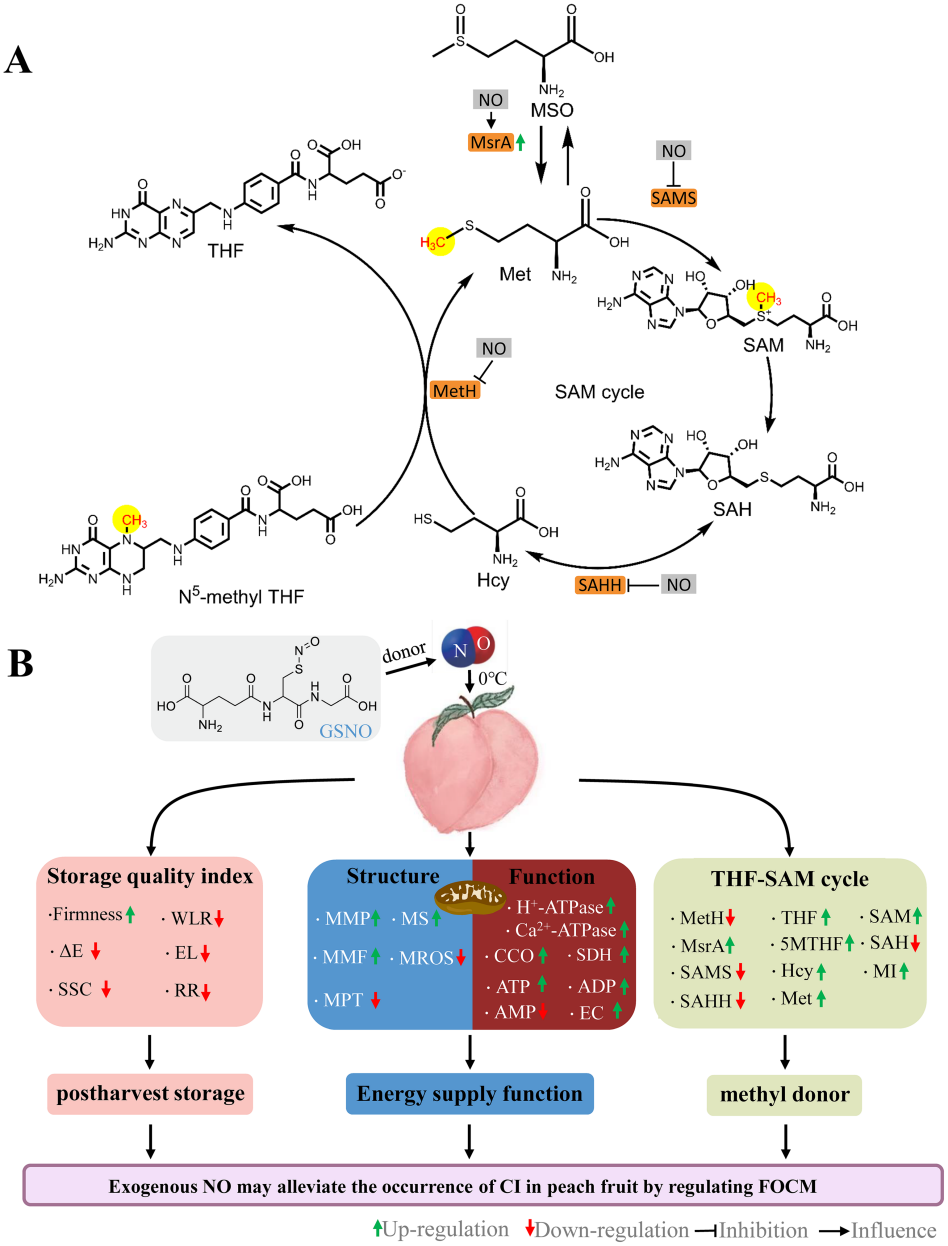
Schematic of the roles by NO on FOCM **(A)** and postharvest storage quality, mitochondrial structural integrity, and mitochondrial energy metabolism function **(B)** in peaches stored at 0°C. GSNO, *S*-nitrosoglutathione; NO, nitric oxide; ΔE, total color difference; SSC, soluble solids content; WLR, weight loss; EL, electrolyte leakage; RR, respiration rate; MMP, mitochondrial membrane potential; MMF, mitochondrial membrane fluidity; MPT, mitochondrial permeability transition; MS, mitochondrial swelling; MROS, mitochondrial ROS; CCO, cytochrome c oxidase; SDH, succinate dehydrogenase; ATP, adenosine triphosphate; ADP, adenosine diphosphate; AMP, adenosine monophosphate; EC, energy charge; MetH, methionine synthase; SAMS, *S*-adenosylmethionine synthetases; SAHH, *S*-adenosylhomocysteine hydrolase; MsrA, Methionine sulfoxide reductases A; 5MTHF, N^5^-methyl-THF; THF, tetrahydrofolate; MSO, methionine sulfoxide; Hcy, homocysteine; Met, methionine; SAM, *S*-adenosylmethionine; SAH, *S*-adenosylhomocysteine; MI, methylation index.

In transgenic tobacco, *MfSAMS1* expression is strongly induced by NO and inhibited by c-PTIO (8). Conversely, sodium nitroprusside (NO donor) decreased SAMS enzyme activity in sunflower seedling cotyledons (33). These suggest that NO-mediated regulation of OCM exhibits opposed effects depending on the species and reagent. Compared with the control, NT treatment decreased the 5MTHF, THF, Hcy, Met, and SAM contents, whereas NO treatment had the opposite effect (Figures 6A–E), suggesting that exogenous NO facilitates FOCM flux accumulation in peaches. Studies have shown that high levels of folate and Met are beneficial for postharvest fruits (4, 5, 34). The contents of 5MTHF, THF, and Met (Figures 6A,B,D) effectively increased, whereas ΔE, SSC, WLR, EL, and RR values decreased in NO-treated peaches (Figures 1C–G). Further, NT treatment reversed this effect of NO, indicating that exogenous NO-induced accumulation of FOCM fluxes (5MTHF, THF, Met) was beneficial for maintaining fruit quality (Figure 8B).

Reactive oxygen species is one of the critical parameters for measuring oxidative damage induced by cold stress. The mitochondrial permeability transition pore (MPTP) is a critical pore that controls the membrane permeability of the inner mitochondrial membrane. Excess ROS may directly oxidize sulfhydryl groups in the MPTP complex proteins, leading to the formation of disulfide bonds and inducing the opening of MPTP. A compromised mitochondrial membrane structure is usually characterized by sustained MPTP opening (35). In this study, the MPT of peaches increased continuously during cold storage, and NO treatment always effectively inhibited the increase in MPT (Figure 2C), indicating that exogenous NO could effectively inhibit MPTP opening induced by oxidative stress. Normal MMPs are prerequisites for maintaining mitochondrial oxidative phosphorylation and ATP production and are necessary to maintain mitochondrial function (36). In this study, NO treatment significantly alleviated the decrease in MMP and MMF compared with the control during storage (Figures 2A,B), suggesting that NO could stabilize MMP and protect MMF. FOCM flux addition (folate and Met) directly increased antioxidant enzyme activity and substance content (4–6). Over time, fruits that underwent NO treatment had higher 5MTHF, THF, and Met contents (Figures 6A,B,D) and lower MROS content (Figure 2E) than the control, suggesting that the increase in FOCM flux induced by NO improved the antioxidant capacity. These findings suggests that NO protects mitochondrial structures by mediating FOCM (Figure 8B). NO synthase (NOS) enzyme is a dimer that relies on the essential cofactor BH4 and available substrates to couple the oxidation of _L_-arginine with the reduction of molecular oxygen to produce NO. When the bioavailability of the substrate is limited or when oxidative stress is elevated, NOS dimers destabilize decoupling, leading to ROS production rather than NO. Cold stress may decrease NO bioavailability (37). 5MTHF, THF, and Met indirectly alleviate oxidative damage in humans and *Escherichia coli* by affecting endogenous NO synthesis and bioavailability (37, 38). This indicates that FOCM flux may directly or indirectly improve antioxidant capacity. Similar to _L_-NAME-induced changes, the increase in Hcy is accompanied by a decrease in NO. The specific impact of FOCM flux on NO bioavailability requires further investigation.

Folate-mediated one-carbon metabolism is essential for maintaining normal mitochondrial function (39, 40). Maintaining intracellular ATP content in fruits and vegetables can inhibit cold stress during storage (41). A study on the cold tolerance of kiwifruit after harvest revealed that the energy loss in fruit is serious, and the quality deterioration is more serious (42). H^+^-ATPase, Ca^2+^-ATPase, CCO, and SDH synergistically regulate the energy metabolism of the plant. In this study, the H^+^-ATPase, Ca^2+^-ATPase, CCO, and SDH activities (Figures 3A–D); the contents of ATP and ADP contents; and EC values (Figures 4A,B,D) gradually decreased over time, indicating that cold stress decreased the activities of mitochondrial respiratory enzymes and led to energy loss occurred. Iron–sulfur (Fe-S) clusters are prosthetic groups that cause mitochondrial electron transfer reactions (39). Synthesizing or repairing Fe-S clusters requires folic acid (43). Therefore, the presence of folic acid may affect mitochondrial oxidative respiration. Direct evidence for this has not been reported in plants; however, in humans, folate deficiency affects mitochondrial oxidative respiration (40). Compared with the control, the H^+^-ATPase, Ca^2+^-ATPase, CCO, and SDH activities (Figure 3); 5MTHF, THF (Figure 6), ATP, and ADP contents; and EC values were lower in the NT treatment (Figure 4). The addition of OCM (folate and Met) improves the efficiency of mitochondrial respiration and affects the cell energy metabolism (44, 45). Compared with the control, NO treatment effectively increased the ATP, ADP (Figure 4), 5MTHF, THF, and Met (Figure 6) contents; H^+^-ATPase, Ca^2+^-ATPase, CCO, and SDH activities (Figure 3); and EC values (Figure 4D), indicating that the maintenance of the energy status of peach fruit is closely related to the promotion of FOCM flux (5MTHF, THF, and Met) accumulation by exogenous NO (Figure 8B). ATP c-subunit synthase is modified by lysine methylation to optimize the mitochondrial ATP synthase function. In contrast, adenine nucleotide translocase, the ADP and ATP carrier to the mitochondrial membrane, is compromised in coupling to ATP synthesis upon methylation (46). Diminished methyltransferase activity due to serine starvation,the source of the one-carbon unit in THF, was accompanied by a significant decrease in ATP levels (47). These suggested that altered intracellular ATP levels might be a response to dynamic changes in methylation. Compared with the control, NO treatment effectively increased MI (Figure 6G), indicating that NO might achieve fresh-keeping by promoting the occurrence of the methyltransferase reaction and changing the synthesis of ATP. FOCM plays an important role in maintaining the stability of mtDNA. For example, it is involved in the conversion of deoxyuridine monophosphate, purine and formyl-methionyl-tRNA synthesis (48, 49). The biosynthesis of the mitochondria-encoded oxidative phosphorylation protein and the regulation of the cellular redox state are closely linked to FOCM (49). These may be how FOCM affects the regulation of mitochondrial function (49).

Exogenous NO can affect the activity of key enzymes of FOCM (33, 50, 51). Danishpajooh et al. (50) found that exogenous NO had an inhibitory effect on the activity of MetH. In salt-stressed sunflower seedlings, exogenous NO inhibits SAMS enzyme activity (33). Treatment with sodium nitroprusside (SNP), an exogenous NO donor, alleviates the onset of CI in frozen peach fruit and significantly promotes MSR expression. Adding c-PTIO inhibits the above effects of SNP stimulation (51). Compared with the control, NO treatment significantly decreased MetH, SAMS, and SAHH activities (Figures 5A–C), and increased MsrA activities (Figure 5D) and 5MTHF, THF, and SAM contents (Figures 6A,B,E) in week 5.This suggests that exogenous NO could regulate FOCM flux by downregulating the enzyme activity of MetH, SAMS, and SAHH and upregulating the enzyme activity of MsrA (Figure 8A). How NO regulates FOCM requires further study.

## 5. Conclusion

Exogenous NO promoted the accumulation of the FOCM components 5MTHF, THF, Met and maintained mitochondrial function and energy levels during cold storage. In this process, the SAM content, and MI values were increased by NO. Exogenous NO could regulate FOCM by affecting the activity of the key FOCM enzymes MetH, SAMS, SAHH, and MsrA. Exogenous NO could promote transmethylation activity by inducing FOCM flux accumulation, which might be responsible for fruit preservation (Figure 8).

## Data availability statement

The raw data supporting the conclusions of this article will be made available by the authors, without undue reservation.

## Author contributions

This work was carried out in collaboration between all the authors. ZY, JF, and SZ conceived designed the experiments. ZY, XW, and CC performed the experiments, analyzed the data, prepared figures and/or tables. ZY and DH wrote the original draft. JF, SZ, DH, CC, and XW reviewed and edited the manuscript. All authors contributed to the article and approved the submitted version.

## Funding

This work was financially supported by the National Natural Science Foundation of China (31800581, 32071808), Key R&D Program of Shandong Province (2022TZXD0023).

## Conflict of interest

The authors declare that the research was conducted in the absence of any commercial or financial relationships that could be construed as a potential conflict of interest.

## Publisher’s note

All claims expressed in this article are solely those of the authors and do not necessarily represent those of their affiliated organizations, or those of the publisher, the editors and the reviewers. Any product that may be evaluated in this article, or claim that may be made by its manufacturer, is not guaranteed or endorsed by the publisher.
